# Field application of an indirect gE ELISA on pooled milk samples for the control of IBR in free and marker vaccinated dairy herds

**DOI:** 10.1186/s12917-018-1716-5

**Published:** 2018-12-05

**Authors:** Barbara Colitti, Elvira Muratore, Maria Elena Careddu, Luigi Bertolotti, Bryan Iotti, Mario Giacobini, Margherita Profiti, Chiara Nogarol, Jens Böttcher, Andreino Ponzo, Roberto Facelli, Sergio Rosati

**Affiliations:** 1 Department of Veterinary Science, University of Turin, Largo Paolo Braccini, 2 10095 Grugliasco, Turin, Italy; 2 Istituto Zooprofilattico Sperimentale del Piemonte della Liguria e della Valle D’Aosta (IZSPLV), 12100 Cuneo, Italy; 3 Tiergesundheitsdienst bayern e.V, 85586 Poing, Germany; 4 Azienda sanitaria locale Cuneo (ASL CN1), 12100 Cuneo, Italy; 5 Associazione Regionale Allevatori Piemonte (ARAP), 12020 Madonna dell’Olmo, Cuneo, Italy

**Keywords:** BoHV1, gE ELISA, Pooled milk, IBR control

## Abstract

**Background:**

The aim of the present study was to assess the reliability of a new strategy for monitoring the serological response against Bovine Herpesvirus 1 (BoHV1), the causative agent of infectious bovine rhinotracheitis (IBR). Bulk milk samples have already been identified as cost effective diagnostic matrices for monitoring purposes. Nevertheless, most eradication programs are still based on individual standard assays. In a region of northwestern Italy (Piedmont), the voluntary eradication program for IBR has become economically unsustainable. Being the prevalence of infection still high, glycoprotein E-deleted marker vaccines are commonly used but gE blocking ELISAs are less sensitive on bulk milk samples compared to blood serum.

**Results:**

A recently developed indirect gE ELISA showed high versatility when applied to a wide range of matrices. In this study, we applied a faster, cost effective system for the concentration of IgG from pooled milk samples. The IgG enriched fractions were tested using a gE indirect ELISA for monitoring purposes in IBR-positive and IBR-marker-vaccinated herds. Official diagnostic tests were used as gold standard. During a 3 years study, a total 250 herds were involved, including more than 34,500 lactating cows. The proposed method showed a very good agreement with official diagnostic protocols and very good diagnostic performances: only 37 positive animals were not detected across the entire study.

**Conclusions:**

The results highlighted the ability of the proposed method to support the surveillance of IBR in the Piedmont region, reducing the costs without affecting the diagnostic performances.

## Background

Infectious bovine rhinotracheitis (IBR) is the most severe disease caused by Bovine Herpesvirus 1 (BoHV1), an etiological agent that is also responsible of other clinical forms such as infectious pustular vulvovaginitis (IPV), abortions and infectious pustular balanophostitis (IBV) [1, 2]. The reactivation from the ganglia of latently infected cattle and re-excretion of BoHV1 results in infection of susceptible or even vaccinated hosts. Control measures for IBR are justified by the substantial economic losses due to the within-farm persistence and to the trade restrictions imposed by IBR-free countries [3].

Several conventional (whole-virus strains) and glycoprotein E-deleted (gE) marker-vaccines are available to contain the clinical forms and reduce the spread of BoHV1. In parallel gE companion diagnostic tests were developed. The gE blocking ELISAs detect antibodies against the missing antigen, differentiating infected from vaccinated animals (DIVA). The use of gE blocking ELISA on milk is discouraged because the level of IgG is about 1/15th of that found in serum and sensitivity issues are not overcome even if un-diluted milk is tested [4–6]. However, milk can easily be collected, pooled and tested, thereby reducing the total costs of surveillance as well as animal stress. According to EU Regulation (2004/558/EC), some prerequisites of BoHV1-bulk-milk testing have to be met: pools must include no more than 50 individual milks (PM) and at least the 30% of cattle on premises must be lactating; finally, the pool size is calibrated according to the limit of detection (LOD) of the method. Whole-virus ELISAs were demonstrated fit for monitoring purposes in IBR-free areas, where vaccination is forbidden. Nevertheless, if adopting a PM approach in marker-vaccinated (MV) farms only DIVA tests could be employed, and blocking ELISAs could lack in sensitivity and specificity [6, 7]. Concentration and purification of IgG from milk by affinity chromatography (AC) partially improved the LOD of blocking gE assays [8]. This approach is however discouraged by the costs and the complexity of the AC protocol. An alternative to AC could consist in testing the IgG-enriched fraction, using cheaper chemical reagents with no adverse effects on ELISA testing.

An indirect ELISA for the detection of IgG against the gE of the BoHV1 was developed in previous studies [9, 10] and showed good performance on serum, individual milk, and purified/concentrated IgG from bulk milk (BM) samples. Since the LOD of the method in concentrated BM was estimated to be equal to a prevalence of 2.5% infected animals per pool, it could be reliable also in MV farms monitoring.

In Piedmont, a voluntary eradication program is currently highly demanding in terms of human and material costs and a further step towards a compulsory eradication program would be unsustainable. This surveillance system is currently operating only in IBR-MV and IBR-free herds, but by changing from blood serum to PM based monitoring, substantial resources could become available for a larger number of farms. Moreover, animals belonging to IBR positive farms are tested only for trade purposes. This aspect leaded to an inaccurate definition on their IBR status at the begin of the study. This lack of information was overcame during the study, updating the official status of all the included farms.

In the present study, individual milk samples, collected for functional feature analysis, were used for PM preparation and an integrated system of partners involved in a cost effective IBR surveillance study was established. Samples were treated with an ammonium-sulphate based concentration protocol, replacing the more expensive AC method. The concentrated PM samples, collected from IBR-positive and IBR marker-vaccinated-farms were tested in gE indirect ELISA. The aim of this study was to evaluate the field diagnostic performance of the method in comparison with the standard individual tests employed in IBR surveillance.

## Results

In the present study, approximately 65,000 individual milks were used for the preparation of isovolumetric pooled milk samples. The mean number of cows per pool was 35 (each pool size ranged from 20 to 40 animals). Approximately 34,500 lactating cows were involved in at least one pooled milk investigation, while 1500 animals (approximately 4%) were excluded for dry period or for treatment with antibiotics. In accordance with EU Decision 2004/558/EC, the aforementioned 1500 animals were individually tested by standard assays. All the individual tests confirmed the previous status of each animal.

**Fig. 1:**
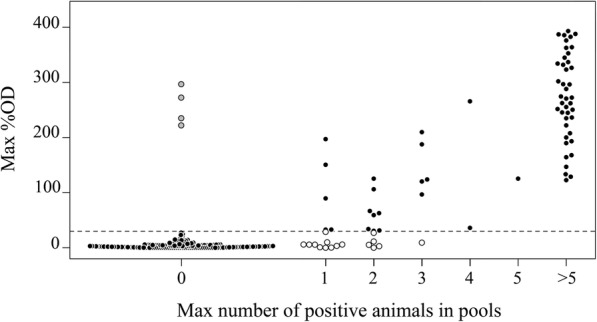
Summary of ELISA Results. All the farms are showed: the position in the plot is given by the maximum number of gE (glycoprotein E) positive animals identified into the pools (x axis) and on the maximum ELISA results recorded among all tested pools. Black dots represent the results in agreement with Official IBR status; grey dots represent the 4 recorded IBR re-infection in expected gE-negative farms; white dots represents the 16 expected gE-positive farms which always resulted negative to gE indirect ELISA. The dotted lines indicate the threshold values of the gE indirect ELISA

Twenty-seven farms were excluded from data analysis since the serological status of the lactating cows was not up-to-date and the estimation of within-pool prevalence could not be performed. Conversely, the IBR status of the remaining 253 recruited farms was updated, permitting the assessment of the diagnostic performance of the method. The updating of the official status allows the number of expected gE-negative farms to increase from 120 to 156, while the number of expected IBR-positive herds was reduced to 97. Description of the farms IBR status is reported in Table 1. In Fig. 1 each farm is depicted according to the highest percentage of OD detected in pooled samples across the entire study (y axis) and the maximum number of expected positive animal per pool (x axis).

**Table 1:** Description of IBR status of the investigated farms according with the presence or absence of antibodies against the glycoprotein E (gE). Thirty involved farms (unknown status) have been never investigated for BoHV1 (Bovine herpesvirus 1) antibodies prior the present study

Farms	May 2015^1^	November 2016^2^
gE-positive	152	97
gE-negative MV	98	156
Unknown status	30	0
Total	280	253

^a^Marker-vaccinated farms. The number of farms belonging to each group at the beginning (1) and at the end (2) of the study is reported

**Fig. 2:**
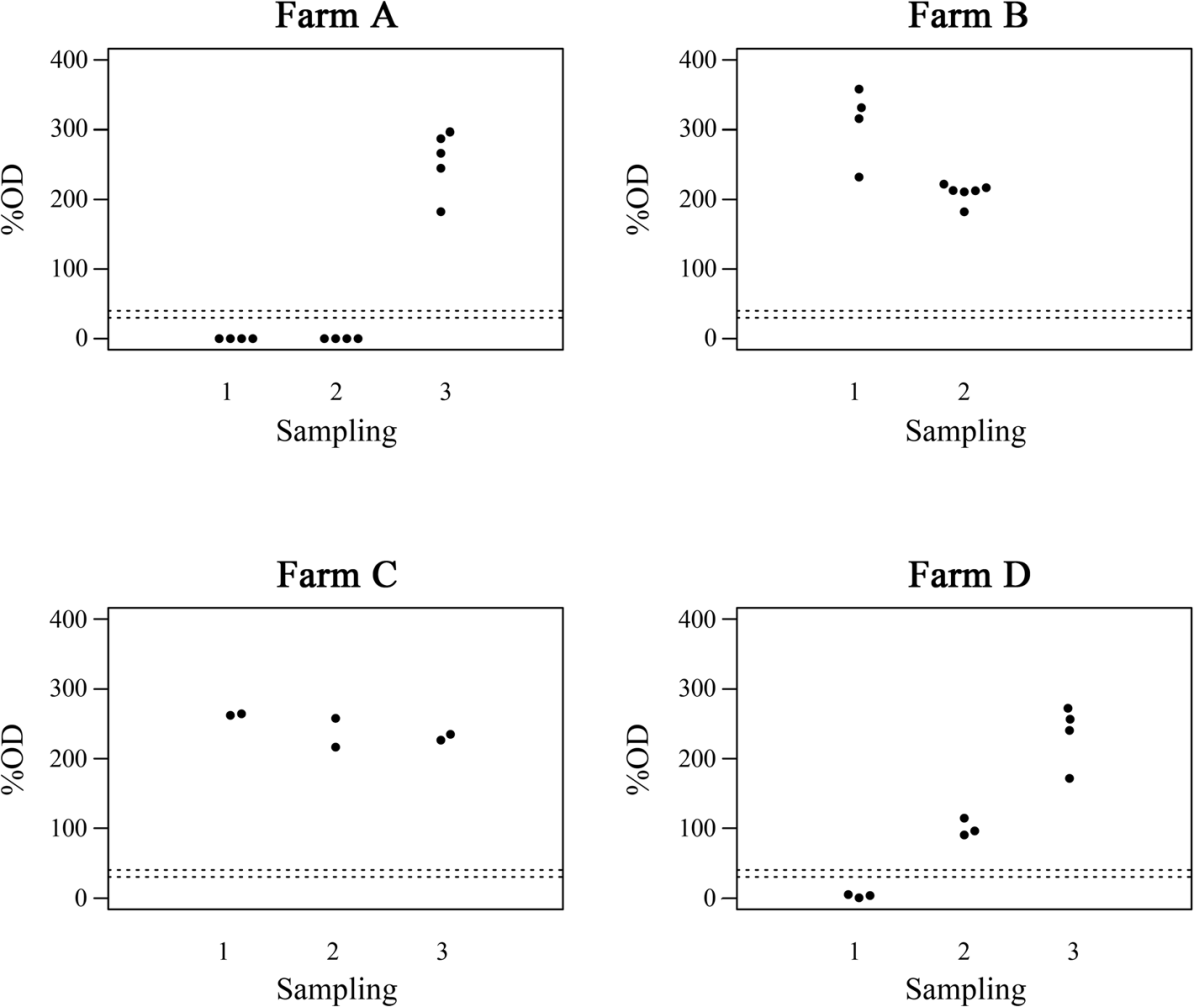
Distribution of IgG enriched fractions ELISA optical density (%OD) related to two/three sampling in the four farms (**a**, **b**, **c**, **d**) with new BoHV1(Bovine Herpesvirus 1) outbreaks detected across the 18 months of the present study. The dotted lines indicate the threshold values of the gE (glycoprotein E) indirect ELISA

The estimation of the diagnostic specificity (DSp) was conducted on the results on all the pools including only gE negative animals belonging to farms with both IBR negative and positive farms status. Among all expected negative pools (*n* = 1224), only 30 resulted in ELISA positive outcome (diagnostic specificity at pool level dSp = 97.55, 95%CI: 96.52–98.43%; at farm level dSp = 95.91, 95%CI: 96.15–100.00%). All of them belonged to four gE negative farms. Further investigations revealed a recent BoHV-1 wild type circulations, promptly detected by pools testing (Fig. 2). The outbreaks were confirmed by the gE blocking ELISA applied to serum samples of the lactating cows. Considering the aforementioned outbreaks, the final evaluation of the DSp at pool level was calculated as 100% (95%CI: 99.69–100.00%). Considering only gE negative farms, the dSp was 100% (95% CI: 96.15–100.00%).

Out of the 97 expected gE-positive farms, 81 confirmed their status in gE indirect ELISA performed on IgG enriched fractions while 16 resulted negative across the entire study, suggesting a raw estimation of the diagnostic sensitivity equal to 83.5% (95% CI: 74.6–90.3%). All the 16 false negative farms were sampled 2 or 3 times and the maximum number of expected positive animals was recorded for each pool. The maximum number of within-pool gE-positive animals ranged from 1 to 3 cows (Fig. 1). A deeper investigation on those farms revealed that 37 gE-positive animals were included in those false negative pools. All the 37 animals have updated IBR statuses, confirming their gE positive status and were 3 to 12 years old (median = 5). Analyses conducted at farm level indicated a very good agreement between the official status and the ELISA results, showing a Cohen’s Kappa equal to 0.8619 (95% CI: 0.7971–0.9268).

## Discussion

Bulk milk or pooled milk testing is a valuable tool for eradication and/or monitoring programs, where a large number of cattle must be tested without inducing stress on the animals and incurring in high costs [11, 12]. Nevertheless, the size of pooled milk samples, in accordance with Decision 558/2004/CE, must be tailored to the LOD of the serological method, ensuring the detection of a single weak positive milk diluted in a modulated pooled milk.

The whole-virus ELISAs have been demonstrated the most sensitive tool for the detection of antibodies against BoHV1 in bulk milk samples [6]. However, in farms where marker vaccination is adopted, whole virus ELISAs fail to distinguish infected from vaccinated cows, and only gE assays are suitable to monitor MV-herds. In the last two decades, the only available tests for IBR in MV-herds were gE blocking ELISAs, which were shown to be less sensitive when applied to PM samples than on individual blood samples (OIE Terrestrial Manual 2010, [6]). Indeed, previous studies demonstrated the importance of the within-pool prevalence and of the stage of lactation (variable levels of IgG) in the sensitivity of gE conventional assays [13]. Nevertheless, new strategies can be adopted to increase sensitivity in BM or PM serological investigations [14] as well as in the surveillance plan strategies [15]. In a recent study [10] we described a new approach for IBR surveillance, based on the use of a novel gE indirect ELISA applied to concentrated/purified BM samples. The concentration protocol consisted in the use of an affinity matrix to bind the IgG that are present in the whey and that can then be purified with a spin-column system.

In the present study, we used the individual milk samples collected for routine functional feature evaluation and identified by a barcode for the preparation of isovolumetric PM samples (1 ml/head). Therefore, this approach bypasses the differences in daily milk production that are often observed when using bulk tank milk. Moreover, the planning of 2–3 sample collections across 18 months reduced the number of animals that were never included in PM investigation to 1500 cows (dried cows and bulls, namely 4.35% of the total), and these had to be tested by individual standard assays.

The diagnostic specificity evaluation involved the 156 gE-negative farms, tested in two independent sample collections and using the gE indirect ELISA applied to IgG enriched fractions. Across the large number of PM samples tested, four cases of BoHV1-seroconversion were promptly detected in the group of gE-negative herds. Indeed, gE blocking ELISA applied to serum samples collected from the four farms confirmed the presence of gE specific antibodies. Even if marker vaccination was in place in 3 out 4 of the new infected herds, the method was demonstrated fit for the purpose of surveillance.

Regarding the 97 expected IBR-positive farms, 81 confirmed their actual IBR status in the outcomes of gE indirect ELISA, while 16 resulted negative. In the current regional intervention program, recognized IBR-positive farms are excluded from yearly monitoring and their official status can stay unchanged for years, despite the progressive elimination of infected heads. Therefore, the number of expected positive farms changed from the beginning of the study and a substantial number of herds were reclassified as gE-negative. Only 16 farms scored negative for the entire study even though recent individual assays have identified 37 gE positive animals included in the respective pooled samples. Those animals represent around the 2% of all the positive animals (*n* = 1805) belonging to gE-positive farms enrolled in the present study.

The apparently false negative results can be partially justified by reasons other than a possible lack in sensitivity of the proposed diagnostic approach. However this condition has been recorded in very few occasions, at least in the area of study and seems correlated to the use of old conventional vaccines (whole virus vaccine, forbidden since 2003) rather than to true viral circulation. Moreover we can not exclude that those animals showed an antibodies titer in the milk samples lower than the limit of detection of the proposed method. On the other hand, new cases of infection were promptly detected and tended to show very high reactivity in all the tested pools regardless of vaccination status. Therefore, the probability of a very small number of truly positive animals being present within the herd without the emergence of an outbreak can be considered quite low. Moreover, the absence of a true gold standard should be taken into account as well as the diagnostic specificity (DSp) value of the commercial gE blocking ELISA applied to serum samples of tested animals. Indeed, the test DSp was measured at 92% [6] and this aspect could, at least in part, justify our results, if we consider these 37 animals (less than one one-thousandth of the total number of heads) as possible false-positive results in gE blocking ELISAs.

## Conclusions

Our findings provide compelling evidence in favor of the reliability a new strategy for IBR surveillance, which includes a traceable and standardized system of PM preparation.

Investigation for IBR surveillance using PM samples could represent a cost-effective solution for monitoring herd-status. Moreover the pooled milk approach can increase the number of serological investigations per year from 1 to 2–3 (according to the herd-qualification), allowing an earlier detection of new BoHV1 outbreaks. We previously demonstrated that the concentration/purification of IgG associated with a gE indirect ELISA represent an effective strategy to overcome the sensitivity issue in BM surveillance. When compared to previous work, in this case we adopted a cheaper, easier and faster protocol for the concentration of IgG from milk, and evaluated its fitness for monitoring purposes. A PM-based surveillance program seems reliable if the pool size is determined accounting for the LOD of the method. Moreover the humoral response against BoHV-1 wild-type strains was found to be high in the target population, regardless the presence of marker-vaccination. The present work was carried out using the preexisting infrastructure for quality assurance programs, which was further improved with a highly automated system of data collection and analysis. The proposed method was proven fit for IBR surveillance in gE-negative farms (DSp of 100%) but also suggested its field applicability for eradication purposes. Indeed, bulk milk samples could be collected from gE-positive farms, processed, and tested until the relative IgG enriched fractions became negative in gE ELISA. This finding will suggest that the within-herd prevalence is low and would justify the adoption of a pooled-milk-based approach. When all pooled milk of those farms test negative, none or only few gE-positive heads are expected to be still in lactation. Therefore, the final step in IBR eradication would consist in the employment of the standard individual assays on blood serum to identify and slaughter the last eventual gE-positive animals. Finally, another advantage of the present study was the active participation of different partners involved in sanitary procedures (ASL and IZSPLV) and functional feature evaluations (ARAP), ensuring the creation of an integrated system with future applicability in different fields of health monitoring in dairy herds.

## Methods

### Farms involved and planning of surveillance

Currently, the regional eradication program in Piedmont includes different levels of IBR status qualifications, based on the herd-immunity against BoHV1. IBR-free farms do not adopt any vaccination program and the entire herd must test negative to whole-virus assays. IBR-marker-vaccinated farms include gE marker vaccinated and IBR-free animals, and new infections can be detected using gE blocking ELISA through individual testing. IBR-positive farms are defined by the presence of antibodies against gE in at least one animal in diagnostic age (> 24 months).

At the beginning of 2015, a group of 280 farms including all IBR official statuses were voluntarily recruited for the present study. All the farms included animals belonging to dairy breeds, in most cases Friesian breed, with a mean size of 80 lactating cows per farm. Milk was collected during the ordinary lactating procedure: this aspect allowed to select only the animals older than 24 months, and, consequently, to exclude the presence of maternal antibodies and colostrum in the analyzed pools.

A total amount of 98 gE-negative IBR-marker-vaccinated farms and 152 gE-positive farms (IBR-positive) were included into two or three PM collection sessions. In addition, 30 farms with unknown IBR status were enrolled in the study and all animals older than 24 months of age were serologically tested using standard assays (SVANOVIR® IBR whole virus ELISA and IDEXX IBR gE Ab blocking ELISA). At the end of the project 254 farms out of 280 were serologically tested using official IBR-ELISAs, updating their IBR status and calculating the within-PM prevalence.

Sample collection was performed across the full 18 months of the study and planned according to the guidelines of Decision 2004/558/EC for IBR eradication programs approved by the European Community: two samples collections were performed in IBR-free and in IBR-MV farms, while three PM preparations, four months apart, were scheduled for the IBR-positive farms.

### Pooled samples: Preparation, identification system and data analysis

Individual milk samples are routinely collected once a month by the Provincial Breeder Association (ARAP) of Cuneo to monitor milk quality through functional feature evaluation. This existing source was used for the preparation of PM samples. From each individual milk sample, 1 ml was collected and used for the preparation of pooled milk that could be processed with the downstream concentration protocol. Each PM sample was identified using a barcode that uniquely identified it and allowed retrieval of the individual ear tags of included animals. Data was stored into a SQL relational database and an ad hoc developed web application was used to interact with the Regional Veterinary database, where diagnostic information is stored by the Official Veterinary Service. This interaction allows retrieval of IBR status (i.e negative, vaccinated or positive) and diagnostic history of each animal included in the pool. This web application also guides the operator in the identification of the optimal pool size (20–40 animals) to optimize LOD based on farm composition.

### Ammonium-sulphate based IgG concentration

Ten ml from each PM were treated with 100 μl of a rennet based solution to precipitate the caseins during an incubation time of 20 min at 37 °C. The obtained curd was broken by manual agitation and incubated on ice for 10 min. The samples were centrifuged at 3600 g for 10 min at 4 °C to obtain three different fractions: a fat layer on top, the whey in the middle and a casein pellet at the bottom of the tube. The whey was transferred to a new 14 ml tube, mixed with an equal volume of a saturated solution of ammonium sulphate (4.1 M at 25 °C) and incubated on a platform shaker for 1 h. After additional centrifugation (3600 g for 10 min at 18 °C), an enriched IgG fraction was visible at the bottom of the tube [16–18]. The supernatant was discarded and the pellet was briefly drained upside down on a paper towel before being resuspended in 400 μl of PBS 1.25% casein. The described protocol allowed a concentration of the milk IgG of about 15X (data not showed). This result is in agreement with previous published data [10]. Even if a different purification method was used, the ammonium sulphate based precipitation method showed a very comparable efficiency. The enriched fractions of IgG were used immediately in the downstream ELISA protocol or alternatively stored at − 20 °C.

### Serological assays

The gE indirect ELISA (ERADIKIT ™ Bulk Milk surveillance Kit, In3diagnostic, Italy), described in two previous papers [10], originally developed for the detection of antibodies in serum or concentrated/purified BM samples, was adapted for application on IgG enriched fractions obtained from PM samples collected in IBR-MV and in IBR-positive farms. The ELISA protocol was slightly modified based on the different diagnostic matrix to be investigated. Briefly, the enriched fractions were placed into two adjacent wells (200 μl/well), the former coated with the recombinant BoHV1 gE, while the latter with a negative antigen. The starting incubation was performed at room temperature (RT) for 120′ minutes. The plate was washed four times and a peroxidase-labeled secondary antibody diluted at 10 ng/ml in PBS 1.25% casein was added to each well. After 45 min of incubation at room temperature, four new washing cycles were performed before the addition of the substrate solution (3,3′,5,5′-tetramethylbenzidine, TMB). The reaction was stopped after 15 min using 100 μl/well of stop solution and read in a microplate spectrophotometer at 450 nm. The reactivity of each sample was calculated as the ratio, expressed as percentage, between the net OD value of the sample (gE-OD value – negative antigen OD value) and the net OD value of the positive control (gE-OD value – negative antigen OD value).

Samples with a percentage of reactivity equal to or greater than 40% were classified as positive, those with reactivity between 30 and 40% as doubtful, and those with a reactivity lower than 30% were considered negative.

### Data analyses

To evaluate the diagnostic performances of the proposed protocol, the ELISA results were compared to the number of positive animals within each milk pool. The goal was to evaluate the ability of the diagnostic protocol to show positive results if at least one gE positive animal was included in the pool. Results of official serological diagnostic procedures were used as gold standard. Diagnostic sensitivity and specificity were evaluated at pool level, as well as at farm level (i.e. all pools collected from a farm during the 18 months surveillance period, fitting the surveillance purpose of the method). The concordance between the results obtained during the study and the official serological investigations was calculated at farm level by Kappa Cohen coefficient. In more details, a farm from which at least one tested milk pool included at least one gE positive animal was considered as expected positive farm. Confidence interval of diagnostic sensitivity and specificity was calculated using Exact Binomial test (R Statistical software ver. 3.2.0).

## Abbreviations

AC: Affinity chromatography
BM: Bulk milk
BoHV1: Bovine Herpesvirus 1
DIVA: Differentiating infected from vaccinated animals
Dsp: Diagnostic specificity
IBR: Infectious bovine rhinotracheitis
IBV`: Infectious pustular balanophostitis
IgG: Immunoglobulin G
IPV: Infectious pustular vulvovaginitis
LOD: Limit of detection
MV: Marker vaccinated
PM: Pooled milk
